# Neuroimaging and analytical methods for studying the pathways from mild cognitive impairment to Alzheimer’s disease: protocol for a rapid systematic review

**DOI:** 10.1186/s13643-020-01332-7

**Published:** 2020-04-02

**Authors:** Maryam Ahmadzadeh, Gregory J. Christie, Theodore D. Cosco, Sylvain Moreno

**Affiliations:** 1 Digital Health Hub, Simon Fraser University, 4190 Galleria 4, 250 – 13450 102 Ave, Surrey, BC V3T 0A3 Canada; 2 School of Interactive Arts and Technology, Simon Fraser University, 250 – 13450 102 Ave, Surrey, BC V3T 0A3 Canada; 3 Science and Technology for Aging Research Institute, Simon Fraser University, 250 – 13450 102 Ave, Surrey, BC V3T 0A3 Canada; 4 Gerontology Research Center, Simon Fraser University, 2800-515 West Hastings St, Vancouver, V6B 5 K3 Canada; 5 Oxford Institute of Population Ageing, University of Oxford, 66 Banbury Road, Oxford, OX2 6PR UK

**Keywords:** Systematic review, Alzheimer, Mild cognitive impairment, Prediction, Conversion, Modality, Neuroimaging, Machine learning, Data analysis

## Abstract

**Background:**

Alzheimer’s disease (AD) is a neurodegenerative disorder commonly associated with deficits of cognition and changes in behavior. Mild cognitive impairment (MCI) is the prodromal stage of AD that is defined by slight cognitive decline. Not all with MCI progress to AD dementia. Thus, the accurate prediction of progression to Alzheimer’s, particularly in the stage of MCI could potentially offer developing treatments to delay or prevent the transition process. The objective of the present study is to investigate the most recent neuroimaging procedures in the domain of prediction of transition from MCI to AD dementia for clinical applications and to systematically discuss the machine learning techniques used for the prediction of MCI conversion.

**Methods:**

Electronic databases including PubMed, SCOPUS, and Web of Science will be searched from January 1, 2017, to the date of search commencement to provide a rapid review of the most recent studies that have investigated the prediction of conversion from MCI to Alzheimer’s using neuroimaging modalities in randomized trial or observational studies. Two reviewers will screen full texts of included papers using predefined eligibility criteria. Studies will be included if addressed research on AD dementia and MCI, explained the results in a way that would be able to report the performance measures such as the accuracy, sensitivity, and specificity. Only studies addressed Alzheimer’s type of dementia and its early-stage MCI using neuroimaging modalities will be included. We will exclude other forms of dementia such as vascular dementia, frontotemporal dementia, and Parkinson’s disease. The risk of bias in individual studies will be appraised using an appropriate tool. If feasible, we will conduct a random effects meta-analysis. Sensitivity analyses will be conducted to explore the potential sources of heterogeneity.

**Discussion:**

The information gathered in our study will establish the extent of the evidence underlying the prediction of conversion to AD dementia from its early stage and will provide a rigorous and updated synthesis of neuroimaging modalities allied with the data analysis techniques used to measure the brain changes during the conversion process.

**Systematic review registration:**

PROSPERO,CRD42019133402

## Background

Dementia causes deterioration of cognitive function that is expected to affect approximately 65.7 million individuals around the world by 2030 [1]. Although there are several types of dementia, Alzheimer’s disease (AD) is the most common type, which comprises around 60-80% of dementia cases [2]. This disease has not only consequences on a social level in terms of costs but also more importantly on patient’s and caregiver’s quality of life [3]. AD can be categorized into three phases: (1) the preclinical phase, which may begin years before diagnosis as AD [4]; (2) mild cognitive impairment (MCI), the transitional stage between normal cognition and AD, during which individuals experience slight impairments in their cognitive function and memory, while maintaining the ability to perform their daily activities [5]; (3) AD, that is indicated by decline in cognitive function and changes in behavior [2]. Although some individuals with MCI remain stable or return to normal cognition, progression to AD occurs at a rate of about 10% to 15% per year [6, 7]. Those cases where individuals convert from MCI to AD are termed progressive MCI (pMCI) and those cases where cognition remains stable are termed stable MCI (sMCI). Currently, it is not entirely understood why some individuals experience pMCI and convert to AD. Therefore, in a clinical context, it is not clear which patients with MCI would benefit the most from clinical interventions and cognitive rehabilitation programs. The ability to predict who will progress to AD dementia from MCI will allow identifying the individuals who could benefit the most from clinical trials. As a result, early prediction of AD could provide the potential therapeutic windows to slow down the progression process [8].

To predict the conversion to Alzheimer’s, researchers have followed several directions. Some studies investigated genetic assessments or biological markers such as those in cerebrospinal fluid (CSF) and many of them explored neuroimaging modalities like electroencephalography (EEG) and functional imaging [9–12]. The main limitation of CSF biomarkers is that their collection requires the invasive procedure of lumbar puncture which is not recommended in routine clinical assessments [13, 14].

Many studies show that AD pathology can be predicted with high accuracy using neuroimaging modalities [15, 16]. These modalities mainly include structural magnetic resonance imaging (sMRI), functional magnetic resonance imaging (fMRI), positron emission tomography (PET), functional single-photon emission computed tomography (SPECT), and electroencephalography (EEG) [16–18]. In each modality, researchers measure the changes of different biomarkers (i.e., changes in gray matter, white matter, or cortical thickness in MRI modality) during the conversion process to predict the conversion from MCI to AD [19, 20]. Both structural and functional magnetic resonance imaging modalities are noninvasive and non-radiation tools for detecting neuronal degeneration at the early stage of AD and investigating progressive brain changes from MCI to AD [21]. Researchers have used PET modalities to investigate metabolic changes during AD progression. In comparison with MRI, PET studies are more invasive and less available, due to their radiation exposure and higher costs [22]. The other type of modality is EEG, which is a non-invasive, extensively available, and low-cost procedure to record the electrical activity of the brain. The findings of a recent study show that EEG coherence is a great predictor of AD progression [23]. Since each modality has its advantages and limitations, it can be expected that the combination of different modalities could provide complementary information [9, 24, 25].

After the identification of informative AD modalities, appropriate data analysis techniques are required to be applied to data to assess the conversion from MCI to AD [26–28]. Recent decades have shown significant growth in the emergence of machine-learning (ML) and pattern-recognition techniques as the effective tools for analyzing brain images to develop the prognosis of AD, while the pathological signs are not observable by visual assessments [5].

In this study, we will conduct a rapid systematic review of the most recent studies that have investigated the progression from MCI to AD dementia using neuroimaging modalities along with machine learning techniques. Thus, the state of the art in neuroimaging and neurodegenerative assessment is presented. There will be two main fields of synthesis in this review. Firstly, we will explore the most recent neuroimaging procedures used to predict the conversion from MCI to AD for clinical applications. Secondly, we will systematically investigate the machine learning techniques applied to create predictive models for the classification of individuals with progressive MCI versus stable MCI.

## Methods/design

### Study design

This is the study protocol for a rapid (systematic) review. A rapid (systematic) review is a form of knowledge synthesis in which components of the systematic review process are simplified or omitted to produce information in a timely manner [29]. This systematic review protocol has been prepared in accordance with the Preferred Reporting Items for Systematic Reviews and Meta-Analyses-Protocols (PRISMA-P) statement [30] (The PRISMA-P checklist is included as Additional file 1). This protocol is registered on PROSPERO (international prospective register of systematic reviews) (registration number CRD42019133402). This review will gather evidence on the effectiveness of neuroimaging modalities and data analysis techniques used to predict conversion from MCI to AD.

### Search methods

We will systematically search the following electronic databases: PubMed/MEDLINE, SCOPUS [31], and Web of Science to provide a rapid systematic review of the eligible studies. We have selected these databases according to preliminary searches and consultation with experts in this field. The reference lists of included articles will be manually searched to identify any missing studies. Keywords related to predicting the progression from MCI to AD will be used. We will use both Medical Subject Headings (MeSH) and keyword searches to increase the sensitivity of the search where possible. Additional file 2 shows the search strategy for PubMed/MEDLINE database.

### Screening and selection procedure

Electronic search results will be downloaded into the Zotero software which is a platform that streamlines the screening of systematic reviews [32]. Two reviewers will independently screen all articles identified from the search. First, all titles and abstracts of the articles returned from initial searches will be screened based on the inclusion/exclusion criteria. Next, full texts will be examined in detail for applicability. If the relevance of an abstract is unclear, it will be reviewed with full-text screening [33]. Any disagreement between reviewers will be resolved by discussion to meet a consensus. A third reviewer will be consulted to decide if consensus is not achieved initially.

### Eligibility criteria

Studies will be included in the review paper, if (1) address research on Alzheimer’s disease dementia and MCI; (2) focus on the prediction of conversion from MCI to AD dementia; (3) explain the results in a way that we would be able to report the performance measures such as the accuracy, sensitivity, and specificity of predicting conversion to AD dementia; (4) describe applied data analysis techniques in sufficient detail to enable replication; (5) use neuroimaging modalities in their research; and (6) are published from January 1, 2017, to the date of search commencement. Since the review paper has focused on a cutting-edge research topic that is fast-paced and developing quickly, we limited the search to examine only the most recent studies, not the outdated ones. Language or publication status restrictions will not be imposed and if necessary, the search will be translated. The unit of assessment will be per-lesion. As the aim of this study is to investigate the transition from MCI to AD dementia, we will exclude articles address research on other types of dementia such as frontotemporal dementia, Lewy body dementia, vascular dementia, Huntington’s disease, and Parkinson’s disease and mixed dementia. Ineligible studies (e.g., conference proceedings, editorial, secondary data analyses, review articles, book reviews) will be excluded as well.

### Data extraction process

We are interested in investigating the studies addressed prediction of progression from MCI to AD dementia using neuroimaging modalities along with machine learning techniques. Thus, we will explore various modalities that have been using in the selected studies as well as data analysis techniques in the early prediction of AD dementia. To achieve this goal, you will develop a bespoke data extraction excel template that captures all the relevant information, and this will be piloted with two independent researchers before it is taken to the full-text extraction. The following information from each included study will be extracted: (1) author(s); (2) year of publication; (3) source of data; (4) follow-up period (conversion period); (5) sample size (i.e., number of participants with stable MCI, progressive MCI ); (7) modalities; (8) neuroimaging features; (9) data analysis techniques, and (10) performance of results in terms of accuracy, sensitivity, specificity, and area under curve (AUC) if available. We will contact authors of primary publications and/or collaborators for clarification if data in an included study is unclear or missing. Discrepancies between reviewers in the extracted data phase will be resolved with discussion. If consensus is not achieved initially a third reviewer will be involved.

### Quality and risk of bias assessment

We will use the quality in prognostic studies (QUIPS) tool to assess the risk of bias (internal validity) of included studies [34]. All articles will be assessed independently by two reviewers within the six domains including study participation, study attrition, prognostic factor measurement, outcome measurement, study confounding, and statistical analysis and reporting. Each reviewer will insert relevant information from each paper in a table that would be rated as high, moderate, or low risk of bias [35]. Discrepant scores will be resolved by discussion or consulting a third member of the group.

### Data synthesis and gap identification

First, the results of the identified studies will be described and summarized in a narrative synthesis. In this context, we will synthesize primary studies to explore heterogeneity descriptively rather than statistically such as structured narratives or summary tables. A descriptive summary will be provided focusing on the investigation of neuroimaging modalities, data analysis techniques, and outcomes. Data synthesis will help us to identify gaps in the evidence, areas of strength, and fields in need of improvement related to methodological development, modalities, and features identification to achieve the main objective of predicting progression from MCI to AD dementia. Second, a quantitative synthesis (i.e., meta-analysis) will be performed if there are sufficient data and if a group of studies is sufficiently homogenous in terms of modalities, data analysis methods, or performance to provide a meaningful summary. We will assess heterogeneity by the Cochran *Q* test and *I*^2^ statistic [36, 37]. For each factor, random effects meta-analysis using the method by DerSimonian and Laird will be conducted to measure the standard mean difference between subjects with progressive MCI and stable MCI. Furthermore, publication bias will be evaluated using visual inspection of the funnel plots and the Egger’s asymmetry test, with *P* values < 0.1 considered significant. For any quantitative analyses, we will conduct sensitivity analyses including a high risk of bias studies. We will use the Grading of Recommendations, Assessment, Development and Evaluation (GRADE) approach to assess the quality of evidence for individual comparisons and outcomes using [38].

## Discussion

This rapid review will provide a knowledge synthesis of the potential neuroimaging modalities as well as data analysis techniques used in recent years to predict the progression from mild cognitive impairment to Alzheimer’s disease dementia. Additionally, we will emphasize the essential need for early prediction of AD to provide the potential therapeutic windows for this progressive disease. Basically, the two main contributions of our review will be as following: (1) an investigation of the recent neuroimaging modalities used for prediction of AD dementia progression, and (2) a discussion of data analysis techniques employed to measure the brain changes during the conversion process. The review will merge different domains of cognitive science, neuroscience, and computer science to benefit the research focused on the prediction of Alzheimer’s disease in the early stage of MCI. The results of the review will identify possible gaps in the current studies and brings knowledge for further research. Any amendments or modifications made in the protocols will be outlined and reported in the final papers. We anticipate a limitation of this study will be a lack of complete and in-depth reporting of biomarkers, data analysis techniques, and data sources in the prediction of AD dementia studies.

Another limitation of this study is that we restricted our search to the studies published from January 1, 2017, to the date of search commencement, which would be a relatively short timeline. The reason behind this consideration is that the review paper has focused on a cutting-edge research topic that is fast-paced and developing quickly, we aimed to examine only the most recent studies, not the outdated ones.

## Supplementary information

**Additional file 1:** PRISMA-P checklist

**Additional file 2:** Key terms for PubMed/MEDLINE search

## Data Availability

Not applicable.

## Abbreviations

AD: Alzheimer’s Disease
MCI: Mild cognitive impairment
PRISMA-P: Preferred Reporting Items for Systematic Reviews and Meta-Analyses extension for Protocols
CSF: Cerebrospinal fluid
SMRI: Structural magnetic resonance imaging
fMRI: Functional magnetic resonance imaging
PET: Positron emission tomography
SPECT: Single-photon emission computed tomography
EEG: Electroencephalography

## References

[1] Prince M, Bryce R, Albanese E, Wimo A, Ribeiro W, Ferri CP (2013). The global prevalence of dementia: a systematic review and metaanalysis. Alzheimer’s & Dementia.

[2] Alzheimer’s Association (2018). 2018 Alzheimer’s disease facts and figures. Alzheimer’s & Dementia..

[3] Dubois B, Hampel H, Feldman HH, Scheltens P, Aisen P, Andrieu S (2016). Preclinical Alzheimer’s disease: definition, natural history, and diagnostic criteria. Alzheimer’s & Dementia..

[4] Sperling RA, Aisen PS, Beckett LA, Bennett DA, Craft S, Fagan AM (2011). Toward defining the preclinical stages of Alzheimer’s disease: recommendations from the National Institute on Aging-Alzheimer’s Association workgroups on diagnostic guidelines for Alzheimer’s disease. Alzheimer’s & Dementia..

[5] Pereira T, Cardoso S, Silva D, de Mendonça A, Guerreiro M, Madeira SC. Neuropsychological predictors of conversion from mild cognitive impairment to Alzheimer’s disease: a feature selection ensemble combining stability and predictability. BMC Medical Informatics and Decision Making [Internet]. 2018 [cited 2019 Aug 11];18. Available from: https://bmcmedinformdecismak.biomedcentral.com/articles/10.1186/s12911-018-0710-y. 10.1186/s12911-018-0710-y PMC6299964 30567554

[6] Petersen RC, Doody R, Kurz A, Mohs RC, Morris JC, Rabins PV (2001). Current concepts in mild cognitive impairment. Arch Neurol.

[7] Ramírez J, Górriz JM, Ortiz A, Martínez-Murcia FJ, Segovia F, Salas-Gonzalez D (2018). Ensemble of random forests one vs. rest classifiers for MCI and AD prediction using ANOVA cortical and subcortical feature selection and partial least squares. J Neurosci Methods.

[8] Facal D, Valladares-Rodriguez S, Lojo-Seoane C, Pereiro AX, Anido-Rifon L, Juncos-Rabadán O (2019). Machine learning approaches to studying the role of cognitive reserve in conversion from mild cognitive impairment to dementia. Int J Geriatr Psychiatry..

[9] Caminiti SP, Ballarini T, Sala A, Cerami C, Presotto L, Santangelo R (2018). FDG-PET and CSF biomarker accuracy in prediction of conversion to different dementias in a large multicentre MCI cohort. NeuroImage: Clinical..

[10] Defrancesco M, Egger K, Marksteiner J, Esterhammer R, Hinterhuber H, Deisenhammer EA (2014). Changes in white matter integrity before conversion from mild cognitive impairment to Alzheimer’s disease. PLoS ONE.

[11] Salvatore C, Cerasa A, Castiglioni I. MRI Characterizes the progressive course of AD and predicts conversion to Alzheimer’s dementia 24 months before probable diagnosis. Front Aging Neurosci. 2018 [cited 2019 11];10. Available from: https://www.frontiersin.org/article/10.3389/fnagi.2018.00135/full. 10.3389/fnagi.2018.00135 PMC5977985 29881340

[12] Zheng L, Kong X, Cui Y, Wei Y, Zhang J, Wei W (2016). Conversion from MCI to AD in patients with the APOE ε4 genotype: prediction by plasma HCY and serum BDNF. Neuroscience Letters..

[13] Liu H, Jiang H, He H, Liu X. A semi-mechanism approach based on MRI and proteomics for prediction of conversion from mild cognitive impairment to Alzheimer’s disease. Sci Rep. 2016 [cited 2019 Aug 11];6. Available from: http://www.nature.com/articles/srep26712. 10.1038/srep26712 PMC4896009 27273250

[14] Riva V, Galloni E, Marcon M, Dionisio L, Deluca C, Meligrana L, et al. Analysis of combined CSF biomarkers in AD diagnosis. Clin Lab. 2014 [cited 2019 Aug 11];60. Available from: http://www.clin-lab-publications.com/article/1469. 10.7754/clin.lab.2013.130440 24779297

[15] Eskildsen SF, Coupé P, García-Lorenzo D, Fonov V, Pruessner JC, Collins DL (2013). Prediction of Alzheimer’s disease in subjects with mild cognitive impairment from the ADNI cohort using patterns of cortical thinning. NeuroImage..

[16] Hojjati SH, Ebrahimzadeh A, Khazaee A, Babajani-Feremi A (2017). Predicting conversion from MCI to AD using resting-state fMRI, graph theoretical approach and SVM. J Neurosci Methods.

[17] Pagani M, De Carli F, Morbelli S, Öberg J, Chincarini A, Frisoni GB (2015). Volume of interest-based [18F]fluorodeoxyglucose PET discriminates MCI converting to Alzheimer’s disease from healthy controls. A European Alzheimer’s Disease Consortium (EADC) study. NeuroImage: Clin.

[18] Suppa P, Kepp T, Lange C, Spies L, Fiebach JB, Dubois B (2016). Performance of hippocampus volumetry with FSL-FIRST for prediction of Alzheimer’s disease dementia in at risk subjects with amnestic mild cognitive impairment. J Alzheimers Dis.

[19] Basaia S, Agosta F, Wagner L, Canu E, Magnani G, Santangelo R (2019). Automated classification of Alzheimer’s disease and mild cognitive impairment using a single MRI and deep neural networks. NeuroImage: Clin.

[20] Cao P, Liu X, Yang J, Zhao D, Huang M, Zhang J (2017). Nonlinearity-aware based dimensionality reduction and over-sampling for AD/MCI classification from MRI measures. Computers in Biology and Medicine..

[21] Fayed N, Modrego PJ, Salinas GR, Gazulla J (2012). Magnetic resonance imaging based clinical research in Alzheimer’s disease. Mandal PK, editor. J Alzheimers Dis.

[22] Musiek ES, Chen Y, Korczykowski M, Saboury B, Martinez PM, Reddin JS (2012). Direct comparison of fluorodeoxyglucose positron emission tomography and arterial spin labeling magnetic resonance imaging in Alzheimer’s disease. Alzheimer’s Dement.

[23] Vecchio F, Miraglia F, Iberite F, Lacidogna G, Guglielmi V, Marra C (2018). Sustainable method for Alzheimer dementia prediction in mild cognitive impairment: electroencephalographic connectivity and graph theory combined with apolipoprotein E: MCI Conversion. Ann Neurol.

[24] Cui Y, Liu B, Luo S, Zhen X, Fan M, Liu T, et al. Identification of conversion from mild cognitive impairment to Alzheimer’s disease using multivariate predictors. Villoslada P, editor. PLoS ONE. 2011 [cited 2019 Aug 11];6. Available from: http://dx.plos.org/10.1371/journal.pone.0021896. 10.1371/journal.pone.0021896 PMC3140993 21814561

[25] Da X, Toledo JB, Zee J, Wolk DA, Xie SX, Ou Y (2014). Integration and relative value of biomarkers for prediction of MCI to AD progression: spatial patterns of brain atrophy, cognitive scores, APOE genotype and CSF biomarkers. NeuroImage: Clinical..

[26] Castiglioni I, Salvatore C, Ramírez J, Górriz JM (2018). Machine-learning neuroimaging challenge for automated diagnosis of mild cognitive impairment: lessons learnt. J Neurosci Methods.

[27] Johnson P, Vandewater L, Wilson W, Maruff P, Savage G, Graham P (2014). Genetic algorithm with logistic regression for prediction of progression to Alzheimer’s disease. BMC Bioinformatics..

[28] Pereira T, Lemos L, Cardoso S, Silva D, Rodrigues A, Santana I, et al. Predicting progression of mild cognitive impairment to dementia using neuropsychological data: a supervised learning approach using time windows. BMC Med Inform Decis Mak. 2017 [cited 2019 Aug 11];17. Available from: http://bmcmedinformdecismak.biomedcentral.com/articles/10.1186/s12911-017-0497-2. 10.1186/s12911-017-0497-2 PMC5517828 28724366

[29] Tricco AC, Antony J, Zarin W, Strifler L, Ghassemi M, Ivory J, et al. A scoping review of rapid review methods. BMC Med. 2015 [cited 2020 Mar 12];13. Available from: http://bmcmedicine.biomedcentral.com/articles/10.1186/s12916-015-0465-6. 10.1186/s12916-015-0465-6 PMC4574114 26377409

[30] Moher D, Clarke M, Ghersi D, Liberati A, Petticrew M, Shekelle P, et al. Preferred reporting items for systematic review and meta-analysis protocols (PRISMA-P) 2015 statement. Sys Rev. 2015 [cited 2019 Aug 11];4. Available from: https://systematicreviewsjournal.biomedcentral.com/articles/10.1186/2046-4053-4-1. 10.1186/2046-4053-4-1 PMC4320440 25554246

[31] Burnham JF. Scopus database: a review. Biomed Digit Libr. 2006 [cited 2019 Aug 11];3. Available from: http://bio-diglib.biomedcentral.com/articles/10.1186/1742-5581-3-1. 10.1186/1742-5581-3-1 PMC1420322 16522216

[32] Trinoskey J, Brahmi FA, Gall C (2009). Zotero: A product review. J Electron Resour Med Libr.

[33] Arksey H, O’Malley L (2005). Scoping studies: towards a methodological framework. Int J Soc Res Methodol.

[34] Hayden JA, van der Windt DA, Cartwright JL, Côté P, Bombardier C (2013). Assessing bias in studies of prognostic factors. Ann Int Med.

[35] Grooten WJA, Tseli E, Äng BO, Boersma K, Stålnacke B-M, Gerdle B, et al. Elaborating on the assessment of the risk of bias in prognostic studies in pain rehabilitation using QUIPS—aspects of interrater agreement. Diagn Progn Res. 2019 [cited 2019 Aug 11];3. Available from: https://diagnprognres.biomedcentral.com/articles/10.1186/s41512-019-0050-0. 10.1186/s41512-019-0050-0 PMC6460536 31093575

[36] Higgins JPT (2003). Measuring inconsistency in meta-analyses. BMJ..

[37] Higgins JPT, Thompson SG (2002). Quantifying heterogeneity in a meta-analysis. Stat Med.

[38] Guyatt GH, Oxman AD, Vist GE, Kunz R, Falck-Ytter Y, Alonso-Coello P (2008). GRADE: an emerging consensus on rating quality of evidence and strength of recommendations. BMJ..

