# Supplementary material for: Neuroimaging and analytical methods for studying the pathways from mild cognitive impairment to Alzheimer’s disease: protocol for a rapid systematic review
Source: Syst Rev. 2020 Apr 2;9:71. doi: 10.1186/s13643-020-01332-7 (PMC7118884; doi:10.1186/s13643-020-01332-7)
Supplement: Supplementary file 2 — Additional file 2: Key terms for PubMed/MEDLINE search [file 13643_2020_1332_MOESM2_ESM.docx]

**Additional file 2**

**Key terms for PubMed/MEDLINE search.**

| **No** | **Search Items** |
| --- | --- |
| **#1** | Alzheimer OR AD |
| **#2** | “Mild cognitive impairment” OR MCI |
| **#3** | predict* OR conver* OR  OR prognos* OR transit* OR progress* OR forecast* OR project* |
| **#4** | neuroimag* OR imag* OR MRI OR “magnetic resonance” OR  fMRI OR “functional magnetic resonance” OR sMRI OR “structural magnetic resonance” OR  PET OR “positron emission tomography” OR SPECT OR “single photon emission computed tomography” OR EEG OR electroencephalogra* OR MEG OR magnetoencephalogra* OR CT  OR “computed tomography” |
| **#5** | Search #1 AND #2 AND #3 AND #4  Limited to studies from 2017 to the date of search commencement. |
